# Associations between long-term exercise participation and lower limb joint and whole-bone geometry in young and older adults

**DOI:** 10.3389/fphys.2023.1150562

**Published:** 2023-05-04

**Authors:** Matteo Scorcelletti, Jochen Zange, Jonas Böcker, Wolfram Sies, Patrick Lau, Uwe Mittag, Neil D. Reeves, Alex Ireland, Jörn Rittweger

**Affiliations:** ^1^ Research Centre for Musculoskeletal Science and Sports Medicine, Department of Life Sciences, Faculty of Science and Engineering, Manchester Metropolitan University, Manchester, United Kingdom; ^2^ Manchester Metropolitan University Institute of Sport, Manchester, United Kingdom; ^3^ Werner Siemens-Endowed Chair for Innovative Implant Development (Fracture Healing), Division of Surgery, Saarland University, Homburg, Germany; ^4^ Institute of Aerospace Medicine, German Aerospace Center (DLR), Cologne, Germany; ^5^ Department of Paediatrics and Adolescent Medicine, University of Cologne, Cologne, Germany

**Keywords:** femur shape, hip shape, master athletes, skeletal development, tibia

## Abstract

**Introduction:** Features of lower limb bone geometry are associated with movement kinematics and clinical outcomes including fractures and osteoarthritis. Therefore, it is important to identify their determinants. Lower limb geometry changes dramatically during development, partly due to adaptation to the forces experienced during physical activity. However, the effects of adulthood physical activity on lower limb geometry, and subsequent associations with muscle function are relatively unexplored.

**Methods:** 43 adult males were recruited; 10 young (20–35 years) trained i.e., regional to world-class athletes, 12 young sedentary, 10 older (60–75 years) trained and 11 older sedentary. Skeletal hip and lower limb geometry including acetabular coverage and version angle, total and regional femoral torsion, femoral and tibial lateral and frontal bowing, and frontal plane lower limb alignment were assessed using magnetic resonance imaging. Muscle function was assessed recording peak power and force of jumping and hopping using mechanography. Associations between age, training status and geometry were assessed using multiple linear regression, whilst associations between geometry and muscle function were assessed by linear mixed effects models with adjustment for age and training.

**Results:** Trained individuals had 2° (95% CI:0.6°–3.8°; *p* = 0.009) higher femoral frontal bowing and older individuals had 2.2° (95% CI:0.8°–3.7°; *p* = 0.005) greater lateral bowing. An age-by-training interaction indicated 4° (95% CI:1.4°–7.1°; *p* = 0.005) greater acetabular version angle in younger trained individuals only. Lower limb geometry was not associated with muscle function (*p* > 0.05).

**Discussion:** The ability to alter skeletal geometry via exercise in adulthood appears limited, especially in epiphyseal regions. Furthermore, lower limb geometry does not appear to be associated with muscle function.

## 1 Introduction

Specific alterations to lower limb bone geometry are important factors that could lead to clinical problems such as fractures (Shin et al., 2017) and osteoarthritis (Do et al., 2020; Scorcelletti et al., 2020). In particular increased femoral torsion, in the order of magnitude of ∼6° can discern between an osteoarthritic and healthy hip in the same subject (Piazzolla, Solarino, et al., 2018) and increases the risk of hip impingement, and knee osteoarthritis (Scorcelletti et al., 2020). Furthermore, Increased lateral femoral and tibial bowing in the order of magnitude of ∼3°–5° are also associated with increased incidence of knee osteoarthritis (Matsumoto et al., 2015; Shimosawa et al., 2018), and femoral and tibial fractures (Chen et al., 2014). Decreased acetabular version in the order of magnitude of ∼2°–6° and femoro-acetabular congruence have been shown to be relevant to hip osteoarthritis, dysplasia and impingement (Tönnis and Heinecke, 1999; Fujii et al., 2010). Osteoarthritis is of particular clinical importance, given that the radiographic evidence shows that the majority of the population over the age of 55 is affected by it (D’Ambrosia, 2005). It is therefore important to identify key factors influencing components of lower limb geometry, and how the latter affects physical function.

Skeletal geometry is not only clinically relevant but also has an effect on movement biomechanics with femoral torsion affecting muscle lever arm length and the line of action of hip muscles (Scorcelletti et al., 2020). Higher femoral torsion (between 21° and 51°) increases internal rotation moment arm length by 96.5% and decreases the external rotation moment arm length by 86%, compared to a general musculoskeletal model (Scheys et al., 2008) likely increasing an in-toeing gait pattern (Scorcelletti et al., 2020). Other hip shape features also have an impact on gait (Lewis et al., 2017) and hip range of motion (Ross et al., 2014). Increased femoral torsion affects kinematics during walking and landing (Scorcelletti et al., 2020), but little is known about how skeletal geometry affects components of physical function such as peak jump power relevant to clinical outcomes as well as sporting performance which could potentially be affected by muscle moment arm length and range of motion.

Lower limb geometry changes dramatically during childhood with femoral torsion decreasing by ∼1.5° every year from the onset of walking until completion of growth is established (Scorcelletti et al., 2020). Varus bowing is also greatest at birth (+15° ± 8), then overcorrects to valgus alignment during the first years of life (−10° ± 8) and stabilizes (−5° ± 8) later in development in healthy children (Salenius and Vankka, 1975). Mechanical loading in early life is a key determinant of bone shape development (Fabeck et al., 2002; Daly, 2007; Janz et al., 2014; Ireland et al., 2019; Saunders et al., 2020) and bone composition (Janz et al., 2001; Daly, 2007; Janz et al., 2014). Individuals who have conditions that do not permit normal gait during development, such as children with cerebral palsy, will often face lower limb misalignments such as increased femoral torsion, tibial torsion, valgus knee alignment and intoeing (Staheli et al., 1968; Fabry et al., 1973; Bobroff et al., 1999; Ward et al., 2006).

Modest changes in lower limb geometry continue to occur throughout adulthood. Bone cross-sectional area increases by ∼15% from 20 years to 90 years (Riggs et al., 2004; Lauretani et al., 2008), whereas femoral torsion also appears to decrease by ∼3° across a similar age range (Waisbrod et al., 2017; Inamdar et al., 2019; Pierrepont et al., 2019). Studies of athletes, who continue to train and compete, have been conducted to examine the effects of physical activity on adult bone cross-sectional geometry. Previous studies have identified greater tibia and femoral cross-sectional area in athletes rather than controls, with larger advantages in athletes competing in high-impact events (Nikander et al., 2005; Wilks et al., 2009a). Similar cross-sectional bone adaptations to exercise have been found in baseball players on the humerus of the pitching arm (Warden et al., 2009). These advantages appear to diminish with age, possibly because of lower muscular forces produced by older athletes (Wilks et al., 2009b). In addition, evidence from master tennis players suggests that the pattern of bone adaptation to exercise differs after skeletal maturity with the ability to adapt bone outer geometry (particularly in epiphyseal regions) substantially diminished compared to adaptations to exercise during development (Ireland et al., 2014).

Whilst the effects of adulthood physical activity on bone cross-sectional geometry have been well explored, comparatively little is known about other clinically relevant aspects of lower limb geometry on the sagittal and frontal plane such as bowing, femoral torsion and acetabular geometry. In the upper limbs, research shows that baseball players have on average 10.6° greater humeral head retroversion (torsion) in the throwing arm (Chant, Litchfield et al., 2007). Femoral bowing might be increased by prolonged squatting tasks or decreased by prolonged heavy lifting tasks due to farming activity in the order of magnitude of 1°–3°(Do et al., 2020), which is clinically relevant for osteoarthritis (Matsumoto et al., 2015; Shimosawa et al., 2018). Therefore, our primary aim was to examine the impact of continued participation in exercise during adulthood on lower limb geometry by comparing young and old sedentary individuals with young and old power athletes. In secondary analyses, we explored associations between femoral and tibial bone shape, and limb alignment on peak lower limb power and peak force during counter-movement jumps and vertical hopping.

## 2 Materials and methods

### 2.1 Study setting

The study was performed between September 2020 and May 2021 at the envihab facility (www.envihab.org) at the German Aerospace Center in Cologne. The study conformed with the declaration of Helsinki. Prior to commencement, it had received ethical approval (Ärztekammer Nordrein, lfd. Nr. 2018269) and had been registered at the German clinical trials register (www.drks.de, registration number DRKS00015764. Recruitment focused on healthy adult males, considering age and training status of the participants. The inclusion criteria for this study were to be either 20–35 years of age (young group) or 60–75 years of age (old group) and male. The participants were selected from individuals either training ≥4 h per week and participating in track and field competitions including sprinting, jumping, and throwing events (athletic, Table 2) or reporting ≤25 metabolic units of physical activity per week (sedentary). The body mass index for all participants was ≤25 kg/m^2^. Combined groups of 24 individuals per age and training category (young/old, trained/untrained) would give 80% study power to detect a large (0.8SD) difference between groups.

Initially, recruitment was planned for spring 2020, which had to be postponed due to the SARS-CoV-2pandemic. Recruitment was challenging due to travelling restrictions and suspected SARS-CoV-2 infections, and candidates were screened from the whole of Germany. Recruitment had to stop when the envihab facility became unavailable in June 2021. All participants gave their written informed consent prior to the testing session. Participants and/or the public were not involved in the design, or conduct, or reporting, or dissemination plans of this research.

### 2.2 Magnetic resonance imaging (MRI) scanning protocol

In this study, 5 sets of 52 cross-sectional images each were acquired from the left leg, hip and lower trunk reaching at least from the foot to the mid lumbar spine. Using a 3 T S Biograph MR, the images were recorded by means of a 6-echo DIXON turbo sequence with the following parameters: flip angle 5°, TR 10 m, TE1 1.35 m, TE2 2.64 m, TE3 = 3.69 m, TE4 = 5.22 m, TE5 = 6.51 m, TE6 = 7.80 m, field of view 300 mm × 300 mm, voxel size of 1.17 mm × 1.17 mm x 5 mm. Only images covering the femur and tibia region were evaluated in this study.

The position of the participants was standardized with a foot-holder and soft wedges keeping the feet perpendicular to the table and the malleoli, epicondyles, and trochanter parallel to the table.

### 2.3 Image analysis

**FIGURE 1:**
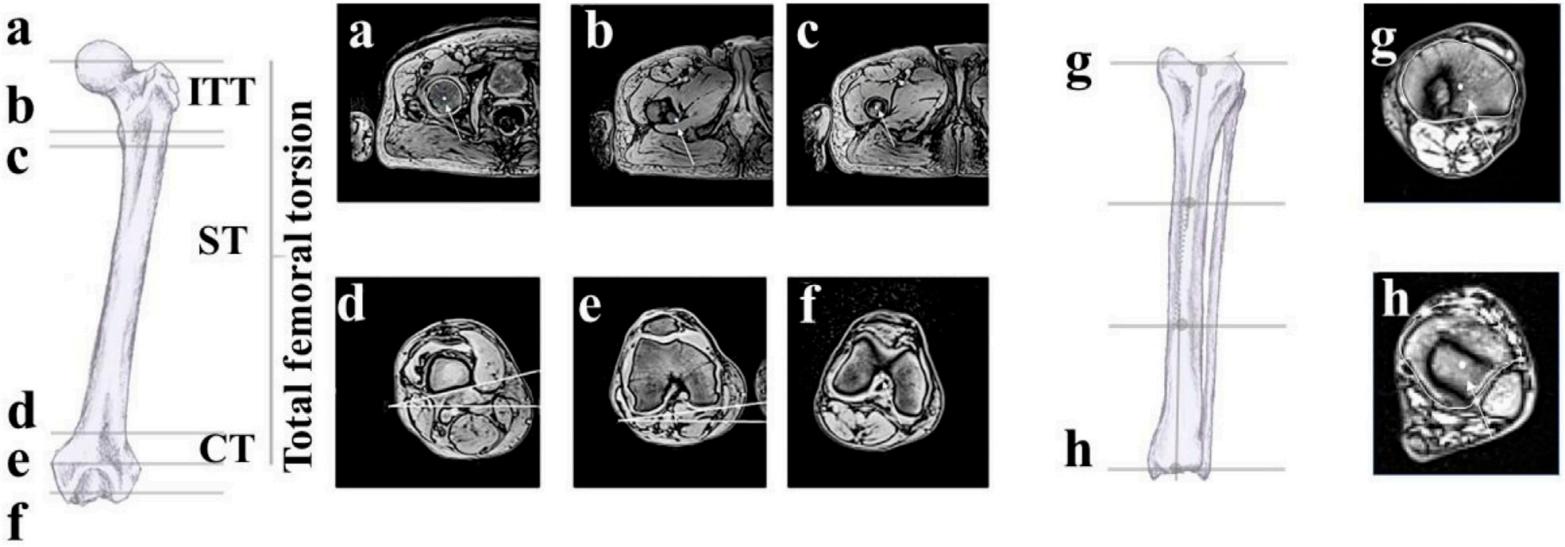
Anatomical landmarks: **(A)** femoral head centre, **(B)** tip lesser trochanter, **(C)** shaft centre, **(D)** posterior distal shaft line, **(E)** posterior condylar line, **(F)** intercondylar notch. The angles measured in “d” and “e” have a horizontal line indicating the coronal plane as reference. Total femoral torsion is the angle between the line joining a and c and the reference in **(E)**. ITT is the angle connecting the landmarks in **(A–C)** and **(B, C)** ST is the angle connecting c-b and the upper line in d, CT is the angle between d and **(C)**. **(G, H)** tibial lateral and frontal bowing landmarks. Figure adapted with permission from Scorcelletti and colleagues, illustrations by Xaali O’Reilly-Berkeley.

MRI images were analysed using ImageJ (version 1.52 h) as in Scorcelletti et al. (Scorcelletti et al., 2022). All the following angles are measured in degrees. Total femoral torsion (also known as femoral neck anteversion) was defined using the femoral neck line and posterior condylar line. The femoral neck line was defined as the line between the center of the femoral head and the center of the shaft directly distal to the lesser trochanter. The posterior condylar line was defined as the line joining the posterior apices of the condyles in the image where the condyles were most prominent. Intertrochanteric torsion (ITT) was defined as the angle between the femoral neck line and the lesser trochanter line. The lesser trochanter line was defined as the line between the apex of the lesser trochanter at the peak of the lesser trochanter size and the center of the femur just under the lesser trochanter. Torsion of the shaft (ST) was defined as the angle between the lesser trochanter line and the distal posterior shaft line, which is aligned with the flat surface of the distal posterior shaft immediately proximal to the posterior condyles. Condylar torsion (CT) is the angle between the posterior distal shaft line and the posterior condylar line (Figure 1). Intra-observer repeatability (assessed as intraclass correlation coefficient–ICC) of total femoral torsion, ITT, femoral lateral and frontal bowing, tibial torsion, tibial lateral bowing, mechanical axis, and femoral mechanical-anatomical angle were excellent (all ICC >0.9). Reliability of CT (ICC0.843), tibial frontal bowing (ICC:0.768), acetabular version angle (ICC:0.859) and acetabular coverage angle (ICC:0.855) measures were good.

To measure the lateral and frontal femoral bowing, the center of the shaft was identified at four landmarks; immediately distal to the lesser trochanter, 1/3 the length of the proximal-distal shaft of the femur; 2/3 of the length of the proximal-distal shaft (the shaft is located between the cuts “c” and “d” in Figure 1) and at the intercondylar femoral notch. Similarly, the lateral and frontal tibial bowing was defined using the center of the shaft at the intercondylar notch, the proximal third of the tibia, the distal third of the tibia at the distal tibial plateau. The two lines defining the mechanical axis angle were those connecting the center of the femoral head and the intercondylar notch, and the intercondylar notch and the center of the distal tibial epiphysis. The two lines defining the femoro-tibial angle were those connecting the centre of the femoral shaft at the distal third of the femur and the intercondylar notch, and the intercondylar notch and the proximal third of the tibia.

**FIGURE 2:**
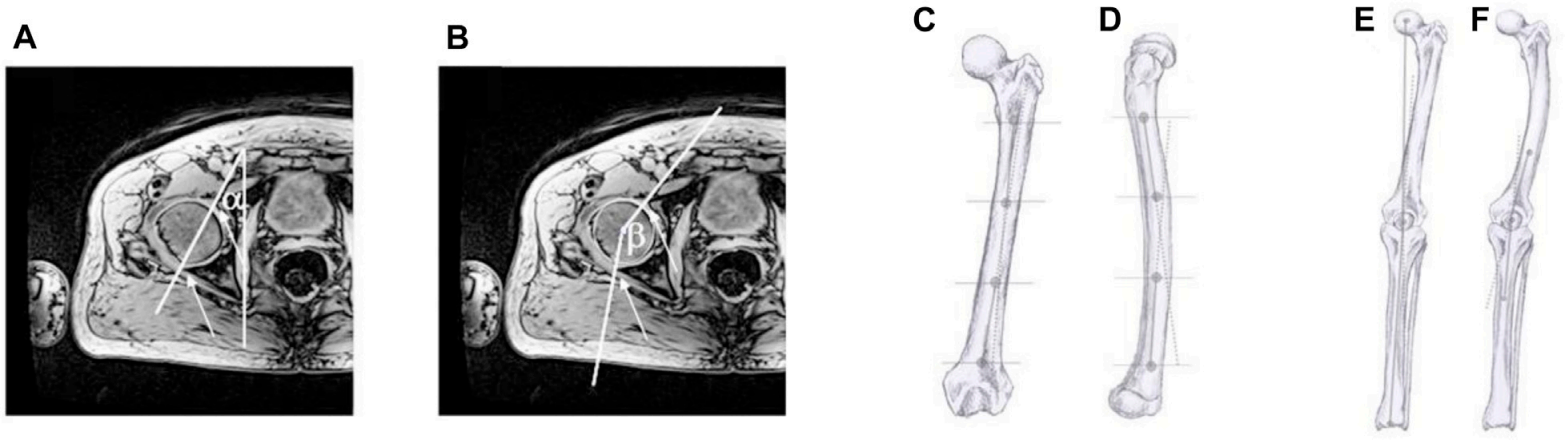
Anatomical landmarks and angles: **(A)** Acetabular version angle *a* and **(B)** acetabular coverage angle *ß*. The slice where the circumference of the femoral head was largest was chosen for analysis. The arrows show the rim of the acetabulum. **(C)** femoral lateral bowing landmarks. **(D)** femoral frontal bowing landmarks. **(E)** mechanical axis landmarks. **(F)** femoro-tibial angle landmarks. Figure adapted with permission from Scorcelletti and colleagues, illustrations by Xaali O’Reilly-Berkeley.

The acetabular version and coverage angle were evaluated in the cross-section where the femoral head was largest. It was defined as the angle between the anterior and posterior acetabular margins of the acetabulum and the sagittal plane (Figure 2). Acetabular coverage was assessed as the angle between the posterior and anterior edge of the acetabulum and the centre of the femoral head.

To calculate the relative angle *λ* in the frontal plane, the coordinates of the desired reference points were calculated using the formula:

$$\lambda =\arctan\left(\frac{\frac{ay-by}{ax-bx}-\frac{cy-dy}{cx-dx}}{1+\frac{ay-by}{ax-bx}*\frac{cy-dy}{cx-dx}}\right)$$

where “a” and “b” represent the proximal and distal reference points of the proximal axis respectively and “c” and “d” the proximal and distal reference points of the distal axis. “B” and “c” overlap in the case of the mechanical axis and femoro-tibial angle (Figure 2). “Y” and “x” on the other hand are respectively the antero-posterior, and the proximal-distal coordinates.

### 2.4 Functional performance

Total relative peak power and force were recorded during 3 countermovement jumps, multiple two-leg hopping, and multiple one leg hopping on a Leonardo mechanography platform (LEONARDO v4.4b01.35). For the countermovement jumps, participants were asked to jump as high as possible, with limited aid from arms swing, with the arms maintained close to the body. For the hopping movements, participants were instructed to bounce on the ball of the foot with stiff knee and ankle joints, with the arms maintained close to the body. They initially began with lower intensity hops, and then over approximately 6–10 hops increased hop intensity until a plateau in peak force was evident. The countermovement jump with the greatest peak power was selected for analysis, and the valid hop with the greater peak force was automatically selected by the software for analysis. Physical activity in metabolic units per week (MET) was assessed using the Freiburg questionnaire of physical activity (FrQ) (Frey, Berg, Grathwohl, & Keul, 1999). The Esslinger Fittnes Index (EFI) is an estimation of the physical fitness level. The results of the counter movement jump are normalized based on gender and age with a score of 100% representing the average value of the German healthy population (Runge et al., 2004).

### 2.5 Statistical analysis

Data were examined using R studio (Version 1.4.1717 2009–2021 Rstudio, PBC, R kernel Version4.1.0). Shapiro-Wilk test was used to test for normal distribution within the age group. Parametric data were analysed within the age group with independent sample Student’s t-test to check for group differences in age, muscle function parameters and physical activity scores between trained and sedentary individuals. Analysis of variance (ANOVA) was used to investigate differences between the 4 age-training groups. Multiple linear regression with age and training status as independent variables, as well as their interaction, was used to determine associations with femoral and tibial geometry, femoro-tibial alignment, and acetabular parameters (Mansournia et al., 2021). Alpha value for main effects was *p* < 0.05, and for interactions *p* < 0.1. Where interaction terms were not evident (beta value *p* > 0.2), the model was simplified to only include main effects. Homoscedasticity of residuals was assessed with the Kolmogorov-Smirnov test. Linear mixed effects models with age and training status as random effects was used to determine main effects of bone geometry on jump and hop outcomes.

## 3 Results

Out of 43 male participants of the study, 22 were considered young (10 trained 24 ± 2.2 years, 12 sedentary 28.8 ± 4.4 years) and 21 old (10 Trained 65 ± 4.1 year, 11 Sedentary 66.9 ± 5.3 years). No differences were evident for height and weight between the 4 groups (Table 1).

**TABLE 1:** Anthropometric data of the cohort. The activity level is based on the Freiburg questionnaire of physical activity. Maximal power relative to body weight has been considered for the countermovement squat jump. Peak force relative to body weight has been considered for the one leg hopping. Age, activity level, relative power and force comparisons are done within the age group. The Esslinger Fittnes Index (EFI) is an estimation of the physical fitness level normalized for sex and age.

		20–34 years old		60–75 years old		Anova
		*10 Trained*	*12 Sedentary*	*10 Trained*	*11 Sedentary*	*p-value*
**Age (years)**	*mean*	24	29	65	67	
	*SD*	2	4 (*p* = .005)	4	5 (*p* = .399)	
**Height (cm)**	*mean*	180.2	181.1	177.6	176.9	0.591
	*SD*	8.3	6.5	7.6	5.6	
**Weight (kg)**	*mean*	77.4	73.6	84.8	79.9	0.655
	*SD*	14	13	33.8	8.9	
**Activity level (MET)**	*mean*	56.6	20.1	94.3	24.8	
	*SD*	21	42 (*p* = .595)	45.4	13.1 (*p* = .047)	
**Relative power(W/kg)**	*mean*	62.5	49.9	42.7	37.0	
	*SD*	6.5	10.0 (*p* = .003)	7.5	7.6 (*p* = .115)	
**Relative Force (N/kg)**	*mean*	33.6	30.9	31.8	24.8	
	*SD*	4.9	3.3 (*p* = .170)	3.3	3.6 (*p* < .001)	
**EFI %**	*mean*	107.3	85.3	112.9	101.8	
	*SD*	8.9	18.0 (*p* = .004)	16.6	20.3 (*p* = 0.201)	

The Athletic group were all competing in track and field power disciplines at regional level or higher including a world record breaker (Table 2).

**TABLE 2:** Discipline and self-reported personal best performances of the athletes. AGP–Age-graded performance, 100%+ world record, 90%+ world class, 80%+ national class,70%+ regional class, 60%+ local class. The percentages have been calculated according to 2010 world records.

Subject N	Young trained (20–34 years old)			Older trained (60–75 years old)		
	*Discipline*	*Personal best performance*	AGP (%)	*Discipline*	*Personal best performance*	AGP (%)
*1*	100 m Sprint	7.0s (60 m)	91	200 m Sprint	27.76s	88
*2*	100 m Sprint	11.63s	84	200; 400 m Sprint	28.64s; 66.30s	90; 89
*3*	100 m Sprint	11.4s	86	400 m Sprint	60.66s	89
*4*	100 m Sprint	11.3s	87	800 m	2min; 24s	91
*5*	100 m Sprint	11.17s	88	60 m; 100 m Hurdles	9.86s (60 m)	92
*6*	100 m Sprint	11.42s	86	Long jump	5.08 m	108
*7*	100,200 m Sprint	NA	NA	Triple jump	11.82 m	92
*8*	400 m Sprint	50.7s	85	Triple jump	10.40 m	79
*9*	Long jump	6.60 m	74	Javelin	36 m	65
*10*	Long jump	NA	NA	Pole vault	2.8 m	78

Training status was associated with increased femoral frontal bowing with the trained group having 2° higher bowing [95% CI: (−3.8°) -(-0.6°); *p* = 0.009] compared to the sedentary group. In addition, a training status by age interaction indicated a larger 4° [95% CI: (−7.1°)- (−1.4°); *p* = 0.005] acetabular version angle in the athlete group only in young individuals. All other parameters were similar between groups (all *p* > 0.13) (Table 3).

**TABLE 3:** Significance (*p*-values) of the multiple linear regression with age and training status as independent variable. When the interaction was evident the *p*-value of the interaction is emboldened and the *p*-value for age and training of the interaction has been reported. Intertrochanteric Torsion (ITT), Shaft Torsion (ST), Condylar Torsion (CT).

Variable	*p*-value		
	*Age*	*Training*	*Interaction*
*Total femoral torsion (°)*	.204	.780	.713
*ITT (°)*	.360	.508	.449
*ST (°)*	.478	.839	.228
*CT (°)*	.454	.544	.638
*Femoral lateral bowing (°)*	.005	.063	.837
*Femoral frontal bowing (°)*	.695	.009	.475
*Femoral mechanical anatomical angle (°)*	.833	.502	.912
*Tibial lateral bowing (°)*	.957	.569	.694
*Tibial frontal bowing (°)*	.997	.917	.273
*Mechanical axis (°)*	.223	.622	.728
*Femorotibial angle (°)*	.178	.670	.817
*Acetabular coverage angle (°)*	.330	.134	.163
*Acetabular version angle (°)*	.847	.005	**.**046

**FIGURE 3:**
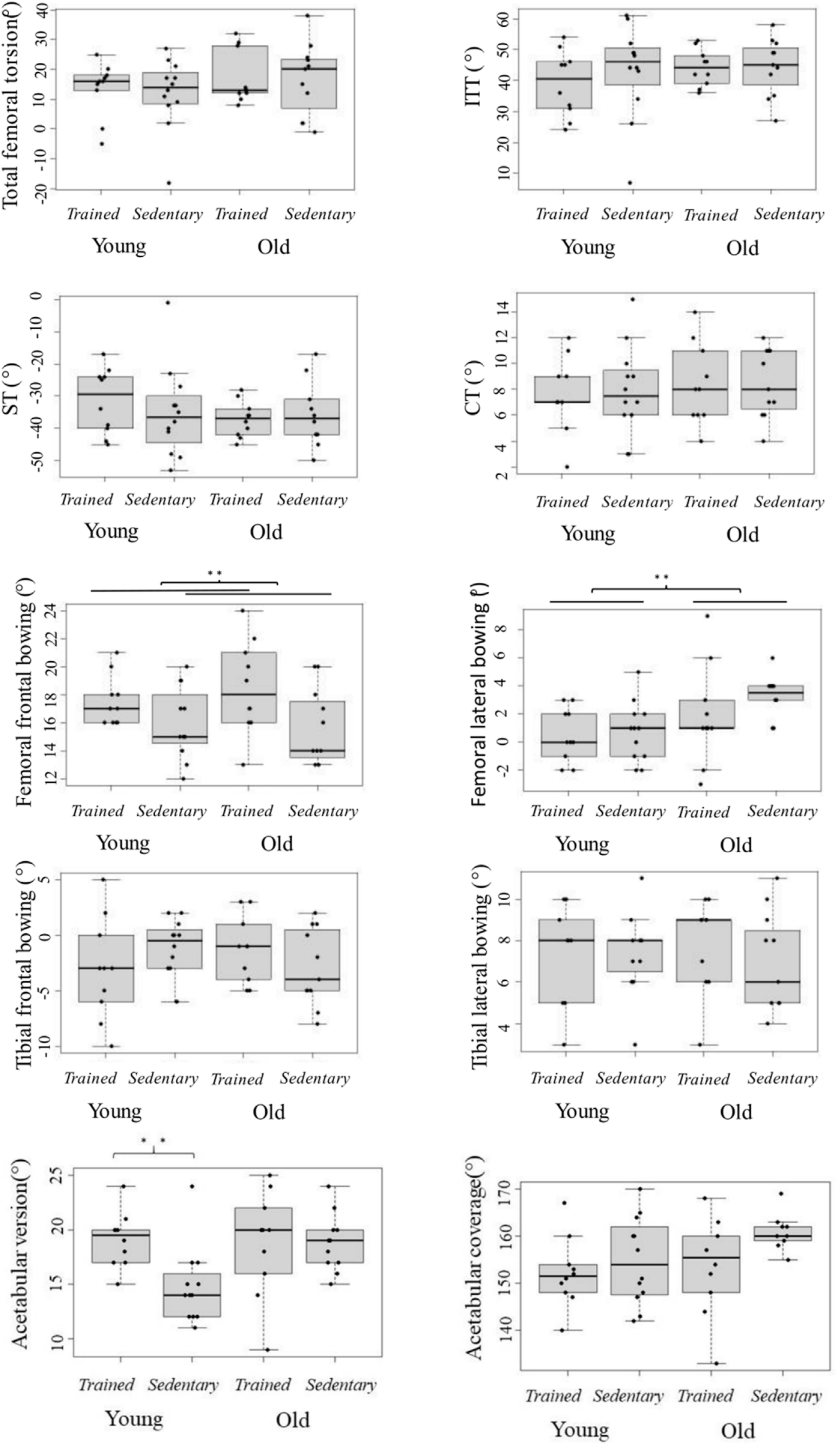
Femoral and tibial parameters grouped into relevant age and training status. ITT is the intertrochanteric torsion, ST the shaft torsion and CT the condylar torsion. Box plots with interquartile range.

Ages showed an association with femoral lateral bowing with the older group having a 2.2° (95% CI:0.8°–3.7°; *p* = 0.01) higher lateral bowing compared to the younger group. Age had no association on all other studied parameters (*p* > 0.17) (Figure 3). Femoral and tibial geometry, and femoro-tibial alignment parameters were not associated with outcomes of countermovement squat jumps, two leg hopping and one leg hopping (Table 4).

**TABLE 4:** Associations between femoral and tibial geometrical parameters and measures of muscle function assessed in this study. In the table we find the estimates as the unstandardised regression coefficients, confidence intervals of the regression coefficients (CI) and significance (*p*-value) of the linear mixed effects. The muscle funtion parameters have been normalised to the participants’ body mass. Relative power has been considered for the countermovement squat jump. Relative peak force has been considered for the two hopping movements. Linear mixed effects model with age and training status as random effects was used to determine main effects of bone geometry on jump and hop outcome. Intertrochanteric Torsion (ITT), Shaft Torsion (ST), Condylar Torsion (CT).

*Variables*	Relative peak power countermovement squat-jump			Relative peak force two leg hopping			Relative peak force one leg hopping		
	*Estimates*	*CI*	*p*	*Estimates*	*CI*	*p*	*Estimates*	*CI*	*p*
Total femoral torsion (°)	−0.06	−0.31–0.19	.632	−0.22	−0.53 – 0.09	.161	−0.01	−0.13–0.11	.852
ITT (°)	−0.09	−0.32–0.15	.483	−0.21	−0.51 – 0.09	.167	−0.06	−0.18–0.05	.298
ST (°)	0.11	−0.14–0.36	.898	−0.9	−2.02 – 0.22	.110	0.04	−0.08–0.16	.496
CT (°)	0.20	−0.70–1.09	.670	0.15	−0.16 – 0.47	.325	−0.33	−0.75–0.09	.134
Femoral lateral bowing (°)	0.44	−0.69–1.57	.214	1.18	−0.19 – 2.56	.090	0.06	−0.48–0.61	.820
Femoral frontal bowing (°)	−0.54	−1.52–0.44	.290	−0.22	−1.48 – 1.03	.722	0.12	−0.36–0.60	.630
Tibial lateral bowing (°)	−1.14	−2.28–0.00	.058	−0.68	−2.21 – 0.85	.374	0.09	−0.49–0.67	.771
Tibial frontal bowing (°)	−0.01	−0.78–0.75	.974	0.02	−0.95 – 0.99	.971	0.14	−0.22–0.51	.447
Mechanical angle (°)	0.58	−0.47–1.64	.283	0.6	−0.76 – 1.96	.382	−0.04	−0.56–0.48	.879
Femorotibial angle (°)	−0.14	−1.17–0.88	.789	−0.6	−1.91 – 0.70	.355	0.02	−0.47–0.52	.932
Acetabular coverage angle (°)	−0.22	−0.48–0.17	.347	0.06	−0.86 – 0.99	.892	−0.07	−0.23–0.09	.338
Acetabular version angle (°)	−0.16	−0.99–0.55	.575	−0.15	−0.56 – 0.27	.474	0.18	−0.18–0.55	.377

## 4 Discussion

This study measured acetabular and lower limb bone geometry, and lower limb alignment in a cohort of young (<35 years) and older (>65 years) track and field power athletes and sedentary individuals. The primary aim was to identify associations between long-term participation in exercise and lower limb geometry in young and older adults. Being an elite track and field athlete was associated with 2° higher femoral frontal bowing regardless of age. In addition, participation in competitive track and field power events, was also associated with age-dependent associations for the acetabular version angle, with the athlete group having a 4° higher acetabular version only in young adults. Age was also associated with femoral shape with the older group having 2.2° higher femoral lateral bowing. Our secondary aim was to identify associations between lower limb geometry and peak power and force output during compound movements such as squat jump and vertical hopping. No lower limb geometry parameter was associated with compound movement performance such as squat jump and vertical hopping.

A number of studies have identified differences in gross lower limb bone morphology between younger athletes and non-athletes, attributable to differences in exercise and associated loading throughout development. Professional ballet dancers had higher neck-shaft angles and lower acetabular version angle than non-athletes (Mayes et al., 2017). Similarly, elite young adult gymnasts had a greater neck-shaft angles than age-match controls (Papavasiliou et al., 2014). No previous studies have examined associations between these measures of bone geometry and clinical outcomes in an athlete population. However, based on studies in the general population we could infer that they are at increased risk of femoral neck fractures in older age (Gnudi et al., 2002). However, studies examining associations between participation in long-term exercise in adulthood and skeletal morphology are scarce. Previous work examining associations of work-related physical activity with femoral bowing found the same magnitude as the current study (Do et al., 2020). Contrary to a study on the humerus of the throwing arm of baseball players, we have found no adaptations to the torsional parameters of the femur (Chant, Litchfield et al., 2007).

Whilst several studies have characterised the dramatic changes in bone morphology which occur during childhood, changes in adulthood remain relatively unexplored. A large study in a Chinese population observed small differences of 1.6° greater neck-shaft angle and lower acetabular version angle in younger adults (<60) compared to older adults (>60 years) (Jiang et al., 2015). However, details of mean age were not given so it is not possible to understand what time gap this association represents. Other studies suggest that femoral torsion decreases during adulthood (Waisbrod et al., 2017; Pierrepont et al., 2019). In contrast, another large study found no difference in neck-shaft angle with age (Gilligan et al., 2013), although this could be explained with variation introduced by the inclusion of samples from a variety of geographical regions and different historical periods. In accordance with our findings, a previous study observed similar age-related differences in femoral bowing (Do et al., 2020).

The absence of pronounced differences in lower limb bone morphology with age and exercise could be related to physeal closure. In previous work, we showed that the mechanoadaptive capacity of bone was markedly reduced when exercise was commenced following skeletal maturity compared to younger starters (Kontulainen et al., 2003; Ireland et al., 2014). In particular, epiphyseal regions seemed to be most affected which may explain the absence of differences in features such as total femoral torsion. In contrast, the diaphysis retained some ability to alter its geometry in response to loading which could account for the small differences in femoral bowing. Indeed, similar effects with age are supported by previous work showing continued reshaping of the periosteal and endocortical borders throughout adulthood (Riggs et al., 2004; Lauretani et al., 2008). This could explain our finding of age-associated differences in shaft bowing, which could arise from remodelling of the diaphyseal borders.

The physiological characteristics of master athletes have been well characterised, and it is clear that regular participation in athletic power events is associated with improved health and function (Tanaka et al., 2011). Further to that, recent longitudinal studies suggest that many of the advantages in indicators of musculoskeletal health such as bone strength and lower limb power are maintained with age (Ireland et al., 2020). The current data suggest that any additional benefits to whole-bone geometry are limited. Whilst greater femoral bowing of ^∼^2° has been found in the trained and in the older group in our study and could be interpreted as a risk factor for osteoarthritis which has been linked to an increased bowing of ∼3°–5° (Matsumoto et al., 2015; Shimosawa et al., 2018). Additionally, the same magnitude of higher femoral bowing evident in master athletes is associated with increased risk of rare atypical femoral fractures (Haider et al., 2019), its relationship with overall fracture risk is unknown. However, importantly these data also do not indicate any negative effects which may negate advantages to other aspects of musculoskeletal health.

Previous literature reported associations between bone shape parameters, neuromuscular function, and biomechanics. For example, total femoral torsion was associated with differences between muscle activation patterns (Nyland et al., 2004), lever arms of the hip muscles (Scheys et al., 2008; Kim et al., 2012; Li et al., 2014) and disadvantageous kinematics during landing (Howard et al., 2011). However, in the current study we did not observe any associations between bone geometry and clinically relevant muscle function outcomes. Muscle function can adapt dramatically in response to physical activity and exercise even in older age (Borde et al., 2015), whereas the current study suggests that the potential for exercise-related adaptations in bone geometry during adulthood is very limited. Therefore, characteristics other than bone geometry may be more important determinants of muscle function in adulthood.

### 4.1 Research implications

Given the greater plasticity of the skeletal system during development, future work should assess associations between bone geometry and muscle function in children and adolescents.

### 4.2 Limitations

As a cross-sectional study, we are unable to attribute causality and it may be that minor associations observed result from differences in other, unmeasured characteristics. Given the evidence of skeletal adaptation to exercise during childhood it may be that differences in early life activity contributed to observed differences and such information would have been difficult to retrieve accurately. Nonetheless, to partially address this concern, Supplementary Material on self-reported metrics such as years of engagement in power disciplines, adherence to structured training regimens, and level of competition during youth have been included. In addition, the study was not powered to detect minor associations of exercise and age with measured geometrical parameters, and may have had reduced power to detect interaction effects. Finally, only males were included to lessen the variability of the measures. A larger secondary study including both sexes, which would be powered to assess group differences despite the increases in variance would be a useful development of this study.

## 5 Conclusion

We observed modest differences in frontal bowing between athletes and non-athletes, and modest differences in acetabular version angle between young athletes and controls. The site-specific nature of these differences may highlight some retained ability to adapt bone shaft geometry in adulthood whereas epiphyseal geometry is largely fixed. Therefore, the ability to alter skeletal geometry via exercise in adulthood appears limited reinforcing the importance of physical activity during development as a strategy to optimise life-long skeletal health. Conversely, skeletal geometry does not appear to be a determinant of muscle function in adulthood. This may in part explain why strategies to improve muscle function remain effective even into old age.

## Data Availability

The raw data supporting the conclusion of this article will be made available by the authors, without undue reservation.
