# Supplementary material for: Associations between long-term exercise participation and lower limb joint and whole-bone geometry in young and older adults
Source: Front Physiol. 2023 May 4;14:1150562. doi: 10.3389/fphys.2023.1150562 (PMC10211427; doi:10.3389/fphys.2023.1150562)
Supplement: Supplementary file 1 [file Table1.DOCX]

|  | Subject  ID | years competing in discipline | Weekly  training hours | Years  following structured training regimen | Competing at national level in Youth |
| --- | --- | --- | --- | --- | --- |
| **Young trained**  **20-34 years old** | 1 | 15 | 18 | 12 | Yes |
|  | 2 | 10 | 10 | 2 | No |
|  | 3 | 8 | 10 | 8 | No |
|  | 4 | 18 | 11 | 5 | No |
|  | 5 | 5 | 10 | 8 | No |
|  | 6 | 0 | 18 | 8 | No |
|  | 7 | 8 | 11 | 8 | Yes |
|  | 8 | 1 | 10 | 10 | Yes |
|  | 9 | 5 | 9 | 8 | Yes |
|  | 10 | 7 | 14 | 4 | No |
| **Older trained**  **60-75 years old** | 1 | 20 | 4 | 20 | Yes |
|  | 2 | 52 | 7 | 45 | Yes |
|  | 3 | 55 | 6 | 55 | Yes |
|  | 4 | 5 | 10 | 5 | Yes |
|  | 5 | 6 | 12 | 50 | Yes |
|  | 6 | 30 | 8 | 40 | No |
|  | 7 | 40 | 8 | 50 | Yes |
|  | 8 | 10 | 14 | 5 | Yes |
|  | 9 | 39 | 5 | 39 | No |
|  | 10 | 6 | 6 | 8 | Yes |

Table 5 presents data pertaining to long-term exercise participation in two age groups: young (<35 years old) and old (60-75 years old) athletic individuals. Specifically, the table reports on the number of years that each participant has competed in their current best discipline, the number of weekly hours spent on training, the duration of following a structured training regimen, and whether the participant had engaged in sports at the national level during their youth, regardless of their current track and field discipline. The preliminary unpublished findings from the TaFMAC study (https://www.dlr.de/me/en/desktopdefault.aspx/tabid-14088/), indicate that less than 20% of master athletes specialize in their latest discipline during their youth. Notably, in their youth, participants typically trained in team sports and achieved a good level of proficiency, as confirmed by our data in the last column of the table. This information serves to provide insight into the exercise habits of athletic individuals across different age groups.
